# Length of hospital stays in patients with psychosis before and after starting on lurasidone

**DOI:** 10.1192/bjo.2021.738

**Published:** 2021-06-18

**Authors:** Uyen Nguyen, Debasis Das

**Affiliations:** Leicestershire Partnership Trust

## Abstract

**Aims:**

This study looked at the differences in the length of hospital stays in number of days, 12 months before and after starting on Lurasidone, in patients with psychosis.

**Method:**

A retrospective review of medical records between 2016 and 2019 of patients with psychosis due to all causes at a First Episode Psychosis service in the United Kingdom was performed. Most common side effects, duration of Lurasidone treatment and reasons for stopping Lurasidone were recorded. The length of hospital stays (in number of days) before and after being started on Lurasidone of those had taken Lurasidone for at least 12 months were compared using a paired t-test.

**Result:**

43 (n = 43) patients had taken Lurasidone at some point during the study period with a mean age of 30.48 years and a male: female ratio of 1.4:1. The average duration of treatment was 327 days. The most common reported side effects were sedation (16%), nausea (7%) and tardive dyskinesia (7%). Among these 43 patients, 19 patients (44%) tolerated and were on Lurasidone for at least 12 months with a mean age of 30.42 and a male: female ratio of 0.42:1. Of these 19 patients, the total number of days of hospital stays within 12 months before and after Lurasidone initiation was 1179 days (mean = 62.05) and 242 days (mean = 16.47) respectively. The paired t-test showed a significant reduction in the average length of hospital stays in these patients within 12 months after Lurasidone initiation (p = 0.0466).

**Conclusion:**

Patients with psychosis who were on Lurasidone had a statically significantly reduction in the length of hospital stays within 12 months of medication initiation; up to 44% tolerance rate, with better tolerance in female patients and the most common side effects being sedation, nausea, tardive dyskinesia.

